# Supplementary material for: Effects of Supplementation with Microalgae Extract from Tetradesmus obliquus Strain Mi175.B1.a on Gastrointestinal Symptoms and Mental Health in Healthy Adults: A Pilot Randomized, Double-Blind, Placebo-Controlled, Parallel-Arm Trial
Source: Nutrients. 2025 Mar 10;17(6):960. doi: 10.3390/nu17060960 (PMC11944429; doi:10.3390/nu17060960)

Supplemental Data for Tetrasol

Supplemental Table S1. Caloric and select nutrient intake at baseline, Week 2/midpoint, and Week 4/final time points.

	TOME			Placebo		
	**Baseline**	**Week 2**	**Week 4**	**Baseline**	**Week 2**	**Week 4**
Calories (Kcal)	1967±125	1870±111	1911±141	2129±119	2124±137	2126±138
Protein (g)	90±7	94±8	85±7	94±7	98±9	99±9
Total fat (g)	84±6	78±6	82±8	90±6	92±8	85±6
Cholesterol (mg)	353±34	355±45	301±29	408±57	410±52	404±60
SFA (g)	26±2	25±2	26±3	29±2	30±3	28±3
MUFA (g)	30±2	27±2	28±3	31±2	31±3	30±2
PUFA (g)	20±2	18±1	20±2	21±2	22±2	19±1
CHO (g)	208±16	199±12	202±16	227±13	219±12	231±17
Fiber (g)	20±3	20±2	20±2	18±1	19±1	19±1
Calcium (mg)	926±72	916±77	947±88	964±91	922±102	964±115
Iron (mg)	14.6±1.5	13.9±1.2	13.9±1.4	14.3±0.83	15.2±1.1	15.8±1.5
Magnesium (mg)	339.5±29.3	322.6±22.1	329.0±29.7	312.2±16.3	305.6±22.3	324.3±24.0
Potassium (mg)	2729.6±224.7	2668.9±174.4	2646.7±187.2	2637.5±167.1	2587.2±191.7	2806.9±273.7
Sodium (mg)	3217.0±210.7	3194.5±207.9	3155.2±218.4	3800.8±241.2	3739.9±298.9	3487.5±221.6
Vitamin K (mcg)	171.6±32.0	141.9±22.7	164.5±30.1	153.8±22.2	139.6±17.5	126.4±21.2
Vitamin D (mcg)	5.3±0.9	4.1±0.5	3.8±0.6	5.3±0.8	3.6±0.5	4.6±0.8
Vitamin E (mg)	10.4±1.2	9.0±0.9	9.4±1.3	9.9±0.8	9.8±1.0	9.0±0.8
Vitamin B-12 (mcg)	5.7±0.9	4.8±0.6	5.1±0.9	5.2±0.7	5.1±0.6	4.9±0.7
Beta-carotene (mcg)	4098±1207	2198±375	3121±540	2573±390	2220±462	2356±599
Vitamin C (mg)	77.9±10.7	73.9±7.5	87.7±12.4	74.6±9.9	68.8±7.8	82.7±11.9

Data are mean ± standard error of the mean (SEM). Abbreviations: CHO, carbohydrates; MUFA, monounsaturated fatty acids; PUFA, polyunsaturated fatty acids; SFA, saturated fatty acids; TOME, *Tetradesmus obliquus* microalgae extract.

Supplemental Table S2. Blood comprehensive metabolic panel.

					Weeks				
**Variable**	**Group**	**N**	**Baseline**		**2**	**4**		**Effect**	**p-Level**
Blood Urea Nitrogen	Placebo	26	13.31 ± 3.65		13.54 ± 3.65	13.42 ± 3.64		Group	0.725
(mg/dL)	TOME	27	12.82 ± 3.28		13.44 ± 2.68	13.11 ± 3.78		Time	0.521
								G x T	0.869
Creatinine	Placebo	26	0.89 ± 0.24		0.88 ± 0.26	0.91 ± 0.23		Group	0.744
(mg/dL)	TOME	27	0.82 ± 0.20		0.79 ± 1.71	1.19 ± 1.71		Time	0.233
								G x T	0.330
Sodium	Placebo	26	140.8 ± 2.4		141.7 ± 2.3	142.2 ± 2.8		Group	0.158
(meq/L)	TOME	27	140.2 ± 2.7		141.1 ± 2.6	141.3 ± 2.5		Time	0.034
								G x T	0.933
Potassium	Placebo	26	4.41 ± 0.40		4.37 ± 0.40	4.34 ± 0.49		Group	0.090
(meq/L)	TOME	27	4.25 ± 0.41		4.16 ± 0.38	4.20 ± 0.44		Time	0.515
								G x T	0.879
Chloride	Placebo	26	108.4 ± 1.6		108.2 ± 1.9	107.9 ± 2.3		Group	0.559
(meq/L)	TOME	27	108.4 ± 1.9		107.8 ± 2.2	107.4 ± 2.5		Time	0.115
								G x T	0.768
Carbon Dioxide	Placebo	26	26.96 ± 1.80		27.42 ± 1.70	26.92 ± 1.89		Group	0.691
(meq/L)	TOME	27	27.44 ± 1.81		27.04 ± 1.74	27.19 ± 1.86		Time	0.822
								G x T	0.401
Calcium	Placebo	26	9.92 ± 0.37		9.90 ± 0.35	9.90 ± 0.35		Group	0.337
(mg/dL)	TOME	27	9.80 ± 0.37		9.80 ± 0.28	9.89 ± 0.40		Time	0.624
								G x T	0.552
Total Proteins	Placebo	26	7.31 ± 0.39		7.28 ± 0.46	7.24 ± 0.36		Group	0.638
(g/dL)	TOME	27	7.31 ± 0.54		7.31 ± 0.47	7.35 ± 0.40		Time	0.948
								G x T	0.559
Albumin	Placebo	26	4.05 ± 0.32		3.97 ± 0.32	3.91 ± 0.20	†	Group	0.786
(g/dL)	TOME	27	3.99 ± 0.24		3.96 ± 0.25	3.93 ± 0.27		Time	0.002
								G x T	0.370
Bilirubin	Placebo	26	1.00 ± 0.42		0.97 ± 0.52	0.93 ± 0.31		Group	0.548
(mg/dL)	TOME	27	0.87 ± 0.38		0.92 ± 0.30	0.92 ± 0.30		Time	0.878
								G x T	0.265
Alkaline Phosphatase	Placebo	26	66.85 ± 11.41	*	65.85 ± 12.87	69.27 ± 11.87	⁑	Group	0.059
(U/L)	TOME	27	57.78 ± 17.47		60.52 ± 16.72	60.52 ± 20.49		Time	0.155
								G x T	0.305
Alanine Transaminase	Placebo	26	26.31 ± 11.40		26.35 ± 12.24	24.46 ± 8.70		Group	0.262
(U/L)	TOME	27	23.78 ± 11.18		21.96 ± 10.16	22.30 ± 9.51		Time	0.260
								G x T	0.501
Aspartate Aminotransferase	Placebo	26	31.46 ± 12.52		27.08 ± 5.53	26.42 ± 5.89		Group	0.882
(U/L)	TOME	27	29.78 ± 9.76		27.82 ± 8.50	26.48 ± 7.56	†	Time	0.002
								G x T	0.537
Glucose	Placebo	26	91.96 ± 6.83		93.08 ± 7.12	94.46 ± 7.51		Group	0.714
(mg/dL)	TOME	27	93.11 ± 8.38		93.19 ± 8.26	95.30 ± 8.87		Time	0.035
								G x T	0.843

Data are expressed as means ± standard deviations. Data were analyzed using a multivariate and univariate General Linear Model with repeated measures (ANOVA). P-levels are listed for between-subject group (G) and univariate within-subject (Greenhouse-Geisser) time (T), and group x time (G x T) effects. Using Tukey's post hoc for multiple comparisons, main effects are indicated by the following superscripts: difference from baseline value, † = p<0.05 (‡ = p>0.05 to p<0.10); and between groups, * = p<0.05 (⁑ = p>0.05 to p<0.10).

Supplemental Table S3: GSRS Scores statistical analysis

				Weeks				
**Variable**		**Group**	**N**	**0**	**2**	**4**	**Variable**	**P-value**
Total score	Raw data	Placebo	*26*	1.88 ± 0.58	1.68 ± 0.54	1.76 ± 0.66 †	Group	0.716
		TOME	*27*	2.12 ± 0.78	1.70 ± 0.43 †	1.64 ± 0.47 †	Time	<0.001
							G x T	0.037
	Baseline adjusted value	Placebo	*26*	*NA*	-0.19 ± 0.35	-0.12 ± 0.37 *	Group	0.035
	(Change from baseline)	*TOME*	*27*	*NA*	-0.42 ± 0.69	-0.48 ± 0.63	Time	0.913
							G x T	0.275
Indigestion score	Raw data	Placebo	*26*	2.27 ± 0.75	2.00 ± 0.82 ‡	2.08 ± 0.97	Group	0.825
		TOME	*27*	2.44 ± 0.96	1.99 ± 0.56 †	1.79 ± 0.69 †	Time	<0.001
							G x T	0.080
	Baseline adjusted value	Placebo	*26*	*NA*	-0.27 ± 0.57	-0.19 ± 0.72 *	Group	0.095
	(Change from baseline)	*TOME*	*27*	*NA*	-0.45 ± 0.91	-0.66 ± 0.84	Time	0.507
							G x T	0.146
Constipation score	Raw data	Placebo	*27*	1.88 ± 0.82 *	1.83 ± 0.79	1.88 ± 1.06	Group	0.047
		TOME	*28*	2.67 ± 1.03	2.12 ± 0.09 †	2.16 ± 1.25 †	Time	0.048
							G x T	0.081
	Baseline adjusted value	Placebo	*27*	*NA*	-0.05 ± 0.63 ⁑	0.00 ± 0.90 *	Group	0.024
	(Change from baseline)	*TOME*	*28*	*NA*	-0.52 ± 1.00	-0.49 ± 1.04	Time	0.744
							G x T	0.951
Diarrhea score	Raw data	Placebo	*26*	2.05 ± 1.14	1.68 + 0.69 ‡	1.91 ± 1.34	Group	0.157
		TOME	*27*	1.89 ± 0.94	1.41 + 0.54 †	1.46 ± 0.54 ‡	Time	0.003
							G x T	0.497
	Baseline adjusted value	Placebo	*26*	*NA*	-0.37 ± 0.85	-0.14 ± 0.85	Group	0.375
	(Change from baseline)	*TOME*	*27*	*NA*	-0.48 ± 0.95	-0.43 ± 1.01	Time	0.241
							G x T	0.446
Abdominal pain score	Raw data	Placebo	*26*	1.86 ± 0.67	1.59 ± 0.66 †	1.65 ± 0.71	Group	0.777
		TOME	*27*	2.09 ± 1.27	1.65 ± 0.62	1.52 ± 0.50 †	Time	<0,001
							G x T	0.192
	Baseline adjusted value	Placebo	*26*	*NA*	-0.27 ± 0.54	-0.20 ± 0.50	Group	0.212
	(Change from baseline)	*TOME*	*27*	*NA*	-0.43 ± 1.06	-0.57 ± 0.95	Time	0.624
							G x T	0.175
Reflux score	Raw data	Placebo	*26*	1.29 ± 0.43	1.29 ± 0.59	1.27 ± 0.45	Group	0.519
		TOME	*27*	1.52 ± 1.04	1.33 ± 0.76	1.30 ± 0.64	Time	0.376
							G x T	0.487
	Baseline adjusted value	Placebo	*26*	*NA*	0.00 ± 0.49	-0.02 ± 0.46	Group	0.352
	(Change from baseline)	*TOME*	*27*	*NA*	-0.19 ± 0.97	-0.22 ± 1.00	Time	0.622
							G x T	0.876

Data are expressed as means ± standard deviations. Data were analyzed using a 2-way mixed-model with repeated measures (ANOVA). P-levels are listed for between-subject group (G) and univariate within-subject (Greenhouse-Geisser) time (T), and group x time (G x T) effects. Using Tukey's post hoc for multiple comparisons, main effects are indicated by the following superscripts: difference from baseline value, † = p<0.05 (‡ = p>0.05 to p<0.10); and between groups, * = p<0.05 (⁑ = p>0.05 to p<0.10). NA means Not Applicable

Supplemental Table S4 : Statistical comparisons of Shannon's Diversity Index values.

Comparison (Shannon's Diversity Index)	Hutchinson T test	P-value	BH adjusted FDR
TOME Baseline vs TOME Week 2	346	0.599	0.641
TOME Baseline vs TOME Week 4	249	0.046*	0.115
TOME Baseline vs Placebo Baseline	185	0.003*	0.041*
TOME Baseline vs Placebo Week 2	189	0.006*	0.045*
TOME Baseline vs Placebo Week 4	205	0.015*	0.071†
TOME Week 2 vs TOME Week 4	287	0.128	0.241
TOME Week 2 vs Placebo Baseline	229	0.019*	0.071†
TOME Week 2 vs Placebo Week 2	229	0.031*	0.093
TOME Week 2 vs Placebo Week 4	242	0.055†	0.118
TOME Week 4 vs Placebo Baseline	293	0.309	0.488
TOME Week 4 vs Placebo Week 2	283	0.326	0.488
TOME Week 4 vs Placebo Week 4	322	0.785	0.785
Placebo Baseline vs Placebo Week 2	294	0.569	0.641
Placebo Baseline vs Placebo Week 4	360	0.519	0.641
Placebo Week 2 vs Placebo Week 4	351	0.464	0.633

P-values based on Hutchinson T-test pairwise comparisons. FDR: False Discovery Rate, calculated using Benjamini-Hochberg correction. *Indicates a significant P-value (<0.05) and † indicates a significant trend (P= 0.051-0.10).

Supplemental Table S5. Saliva and plasma ELISA assays

Variable	Group	N	Baseline			Week 4			P-Value
Saliva cortisol	Placebo	26	3.31	±	1.67	3.33	±	1.36	0.998
(ug/dl)	TOME	27	3.48	±	1.50	3.39	±	1.28	0.926
Serum cortisol	Placebo	25	15.09	±	6.84	15.74	±	7.41	0.834
(ug/dl)	TOME	25	18.07	±	7.82	17.23	±	9.17	0.742
Salivary amylase	Placebo	25	95.81	±	77.12	104.21	±	75.82	0.511
(U/L)	TOME	23	126.22	±	77.00	114.02	±	88.05	0.361
Serum a-amylase	Placebo	25	50.39	±	13.55	45.74	±	16.48	0.090
(U/L)	TOME	25	46.81	±	11.60	44.88	±	12.16	0.640
Saliva CgA	Placebo	25	394.9	±	453.5	467.8	±	559.2	0.516
(ng/ml)	TOME	22	421.5	±	730.4	333.3	±	463.3	0.461
Serum CgA	Placebo	25	1305.9	±	1063.5	1260.5	±	1054.3	0.947
(ng/ml)	TOME	25	1053.6	±	1116.1	677.9	±	598.4†	0.037
Serum BDNF	Placebo	24	15.85	±	12.80	13.76	±	8.34	0.689
(ng/ml)	TOME	26	14.83	±	12.99	11.59	±	8.53	0.389
Serum ATCH	Placebo	24	14.41	±	8.82	16.15	±	11.07	0.269
(pg/ml)	TOME	26	16.14	±	27.58	17.32	±	30.22	0.433

Data are expressed as means ± standard deviations. Data were analyzed using mixed-effects analysis for multiple comparisons. P-values represent comparison from baseline to final for each treatment group. † = p<0.05

Supplemental Table S6. **Brachial and aortic blood pressure and augmentation index (AIx) at rest and during the CPT.**

Outcome	Condition	TOME		Placebo		*p*-Value		
		Baseline	4-Weeks	Baseline	4-Weeks	Time	Tx	Time x Tx
bSBP	Rest	106.17 ± 1.36	106.33 ± 1.36	108.42 ± 2.14	109.65 ± 2.33	0.420	0.233	0.518
	CPT – 1 min	131.30 ± 3.16	132.93 ± 3.61	130.52 ± 2.44	130.79 ± 2.41	0.667	0.678	0.655
	CPT – 2 min	117.85 ± 5.46	123.26 ± 2.77	121.31 ± 2.50	124.12 ± 2.39	0.140	0.627	0.614
	1 min post CPT	111.44 ± 1.95	111.37 ± 1.81	113.73 ± 2.44	115.12 ± 2.55	0.520	0.295	0.478
	2 min post CPT	109.85 ± 1.60	109.11 ± 1.70	110.08 ± 2.02	112.80 ± 2.70	0.329	0.453	0.099
	3 min post CPT	108.96 ± 1.76	107.44 ± 1.25	110.20 ± 2.18	111.0 ± 2.52	0.818	0.366	0.236
bDBP	Rest	65.98 ± 1.36	66.86 ± 1.32	65.46 ± 1.57	67.01 ± 1.33	0.051	0.944	0.550
	CPT – 1 min	90.59 ± 2.64	89.96 ± 3.10	87.24 ± 1.72	88.38 ± 1.53	0.894	0.414	0.578
	CPT – 2 min	74.8 ± 3.71	81.0 ± 2.08*	77.81 ± 1.67	80.48 ± 1.71	0.016	0.712	0.307
	1 min post CPT	69.67 ± 1.92	70.30 ± 1.88	70.04 ± 1.66	71.32 ± 1.51	0.357	0.792	0.799
	2 min post CPT	67.22 ± 1.70	68.15 ± 1.67	67.23 ± 1.71	68.84 ± 1.65	0.197	0.874	0.728
	3 min post CPT	66.96 ± 1.49	67.04 ± 1.57	65.96 ± 1.73	66.88 ± 1.67	0.549	0.745	0.604
aSBP	Rest	98.59 ± 1.42	99.85 ± 1.30	99.35 ± 1.68	102.2 ± 1.51*	0.028	0.404	0.374
	CPT – 2 min	114.67 ± 2.67	117.78 ± 2.36	113.62 ± 2.13	115.08 ± 2.01	0.076	0.523	0.461
aDPB	Rest	67.89 ± 1.18	69.07 ± 1.10	68.27 ± 1.57	70.24 ± 1.21*	0.045	0.622	0.589
	CPT – 2 min	78.19 ± 2.39	79.04 ± 1.77	77.46 ± 1.71	78.76 ± 1.52	0.269	0.839	0.822
aMAP	Rest	79.37 ± 1.34	80.67 ± 1.17	80.19 ± 1.69	82.28 ± 1.31	0.052	0.471	0.608
	CPT – 2 min	91.74 ± 2.54	93.78 ± 1.93	90.73 ± 1.80	92.08 ± 1.61	0.10	0.614	0.739
HR	Rest	60.93 ± 1.67	63.22 ± 1.75	61.42 ± 1.72	61.32 ± 2.17	0.419	0.780	0.457
	CPT – 1 min	70.33 ± 2.09	70.56 ± 2.27	68.64 ± 2.70	66.67 ± 3.07	0.557	0.462	0.431

	CPT – 2 min	59.93 ± 2.08	61.15 ± 1.57	59.42 ± 1.80	60.56 ± 2.30	0.273	0.869	0.938
	1 min post CPT	61.11 ± 2.03	61.56 ± 1.72	59.42 ± 1.65	59.52 ± 2.13	0.710	0.484	0.968
	2 min post CPT	59.85 ± 1.89	60.67 ± 1.78	59.46 ± 1.94	59.12 ± 1.95	0.559	0.796	0.778
	3 min post CPT	60.41 ± 2.04	61.52 ± 1.81	60.44 ± 1.65	59.36 ± 2.03	0.706	0.654	0.465
AIx (%)	Rest	15.37 ± 2.99	14.78 ± 3.05	10.65 ± 2.81	9.52 ± 2.28	0.565	0.195	0.889
	CPT – 2 min	22.81 ± 3.17	25.0 ± 3.04	14.46 ± 2.22†	16.36 ± 2.09†	0.137	0.021	0.996
AIx@75 (%)	Rest	8.56 ± 3.32	9.07 ± 3.13	4.08 ± 3.12	2.92 ± 2.72	0.928	0.213	0.677
	CPT – 2 min	15.48 ± 3.54	18.30 ± 3.13	7.12 ± 2.52	9.40 ± 2.44†	0.097	0.034	0.972

Comparisons were made using a repeated measure two-way analysis of variance (ANOVA) with Tukey's correction. Post hoc significance P < 0.05 *compared with baseline, † compared with Treatment 1. Abbreviations: a, aortic; b, brachial; Tx, treatment.

Supplemental Table S7. Blood lipid panel data.

	TOME			Placebo		
	**Baseline**	**Week 2**	**Week 4**	**Baseline**	**Week 2**	**Week 4**
**Total cholesterol (mg/dL)**	184±33	186±33	184±32	183±30	180±31	182±27
**LDL (mg/dL)**	103±25	103±26	98±24	105±27	102±29	105±26
**HDL (mg/dL)**	63±14	65±15	63±16	60±13	57±13	58±12
**Triglycerides (mg/dL)**	100±45	91±41	116±59*	95±47	94±33	95±51

*Significantly higher than both Baseline and Week 2 values. LDL=low density lipoprotein; HDL= high density lipoproteins.

Supplemental Table S8. PBMC Markers of systemic inflammation

Variable	Group	N	Baseline			Week 4				Effect	P-Value
IFN	Placebo	24	108.4	±	52.1	97.1	±	66.4		Group	0.396
(pg/ml)	TOME	26	115.1	±	64.7	115.2	±	72.7		Time	0.618
										G x T	0.613
IL-2	Placebo	24	16.0	±	18.8	15.8	±	18.5		Group	0.539
(pg/ml)	TOME	26	17.1	±	16.7	20.8	±	21.3		Time	0.367
										G x T	0.304
IL-10	Placebo	24	2456.1	±	922.5	2259.5	±	928.6		Group	0.079
(pg/ml)	TOME	26	1841.9	±	951.8	1981.5	±	1114.2		Time	0.822
										G x T	0.188
TNF	Placebo	24	826.1	±	403.9	914.9	±	536.9		Group	0.702
(pg/ml)	TOME	26	760.7	±	440.3	902.2	±	393.8		Time	0.132
										G x T	0.727
IL-21	Placebo	24	2.45	±	5.25	1.61	±	2.14		Group	0.176
(pg/ml)	TOME	26	1.06	±	0.36	1.37	±	0.78		Time	0.617
										G x T	0.285
IL-5	Placebo	24	9.74	±	29.65	4.51	±	8.52		Group	0.161
(pg/ml)	TOME	26	2.21	±	1.40	3.08	±	3.05		Time	0.459
										G x T	0.303
IL-13	Placebo	24	126.8	±	130.0	106.1	±	110.6		Group	0.176
(pg/ml)	TOME	26	68.8	±	48.1	96.9	±	78.1		Time	0.753
									†	G x T	0.042
IL-17	Placebo	24	18.9	±	18.3	19.8	±	24.7		Group	0.497
(pg/ml)	TOME	26	15.5	±	11.1	17.3	±	9.3		Time	0.494
										G x T	0.829
IL-4	Placebo	24	0.099	±	0.152	0.086	±	0.092		Group	0.876
(pg/ml)	TOME	26	0.092	±	0.101	0.102	±	0.125		Time	0.944
										G x T	0.541

Data are expressed as means ± standard deviations. Data were analyzed using mixed-effects analysis (ANOVA). P-values are listed for treatment group (G), time (T), and group x time (G x T) effects. † = p<0.05

Supplemental Figure S1. Ratios of Abnormal:Total Bowel Movements. A) Ratio of Constipation, defined as BM recorded as a 1 or 2 on the Bristol Stool Scale compared to total bowel movements, including data from all study participants. B) Constipation ratio, including only the data from individuals who reported constipation at Baseline. C) Ratio of Diarrhea, defined as BM recorded as a 6 or 7 on the Bristol Stool Scale compared to total bowel movements, including data from all study participants. D) Constipation ratio, including only the data from individuals who reported constipation at Baseline. No significant differences in bowel habits were observed with either treatment group.

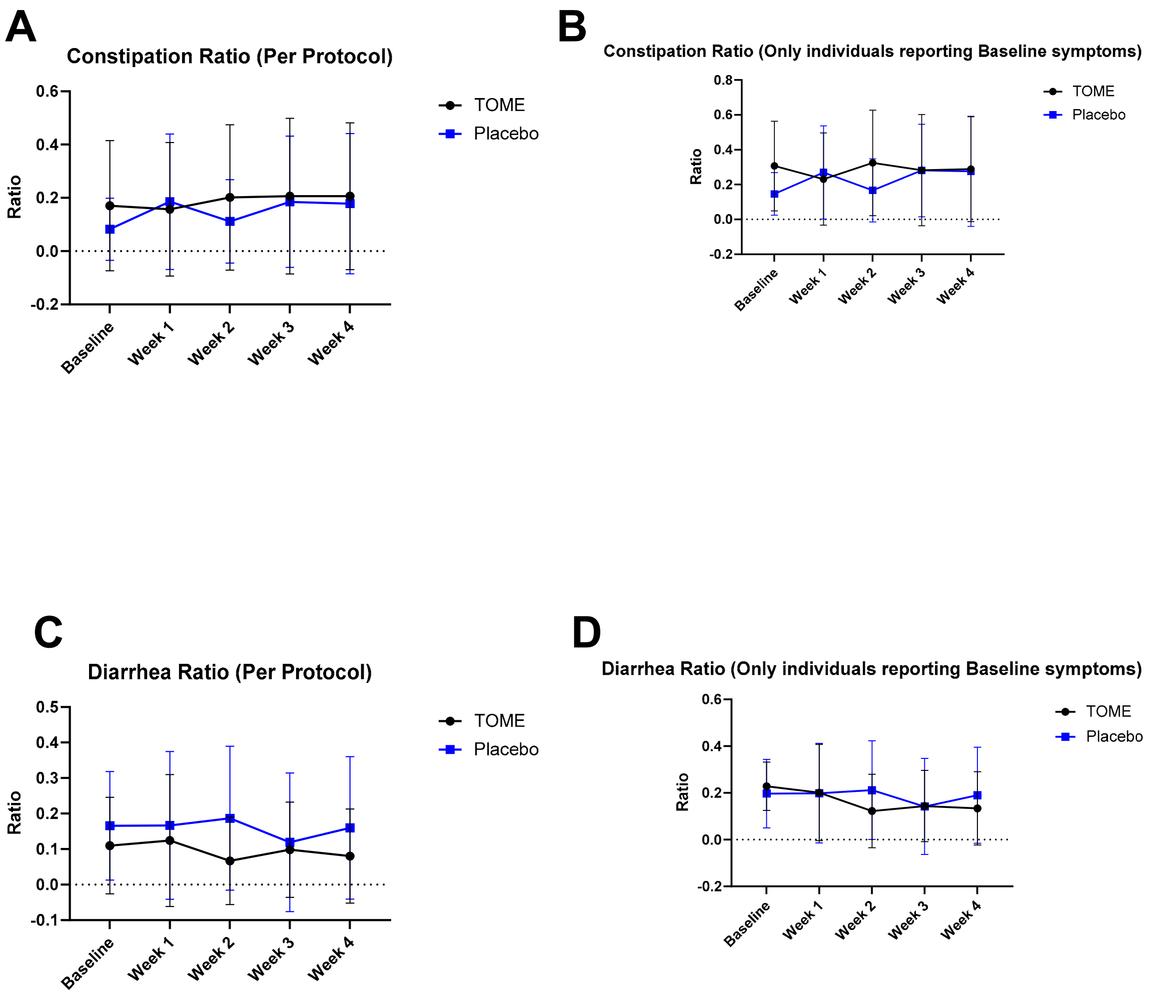

Supplement: Supplementary file 1 [file nutrients-17-00960-s001.zip › nutrients-3489155-supplementary.pdf]
